# Histiocytic Sarcoma of Palatine Tonsil in a Pediatric Patient: A Case Report of a Rare and Aggressive Malignancy

**DOI:** 10.1002/ccr3.72745

**Published:** 2026-05-25

**Authors:** Ihsan ullah, Muhammad Sanaan Noor, Hammad Saif, Muhammad Maaz Bin Zahid, Muhammad Hassan Noor, Muhammad Asim Shah, Aizaz Anwar Khalid, Somaiya Ahmed

**Affiliations:** ^1^ Department of ENT Khyber Teaching Hospital Peshawar Pakistan; ^2^ Department of Medicine Khyber Medical College Peshawar Pakistan; ^3^ Department of Medicine Peshawar Medical College Peshawar Pakistan; ^4^ Department of Internal Medicine Sir Salimullah Medical College Dhaka Bangladesh

**Keywords:** histiocytic sarcoma, immunohistochemistry, palatine tonsil, pediatric oncology, tonsillar neoplasm

## Abstract

A 7‐year‐old boy with a history of recurrent tonsillitis presented with progressive left‐sided throat pain, foreign body sensation, and cervical lymphadenopathy. During the examination, doctors found an ulcerated mass on the left palatine tonsil. A contrast‐enhanced CT scan showed an enlarged tonsil that had uneven growth and narrowing of the opening. The tissue sample revealed large, abnormal cells with a lot of pinkish cytoplasm, irregular nuclei, and a high Ki‐67 index (around 80%). Immunohistochemistry tests confirmed the cells were of histiocytic origin, strongly expressing CD68, CD163, LCA, and S100, confirming the diagnosis of histiocytic sarcoma. The boy had surgery to remove the affected tonsil and lymph nodes, followed by chemotherapy with cyclophosphamide, doxorubicin, vincristine, and prednisolone. This case highlights a rare and unusual form of a serious blood cancer in a child. It also shows the diagnostic difficulties, as benign issues like recurrent tonsillitis can hide more serious problems. Making a correct diagnosis required a timely biopsy and suitable immunohistochemical tests. Given the tumor's high growth rate and risk of spreading early, treatment needed a team approach that involved complete local removal and immediate systemic therapy. Long‐term follow‐up is important, and more case reports and studies are necessary to determine the best treatment options and improve results in pediatric histiocytic sarcoma.

## Key Clinical Message

Histocytoma arising from the palatine tonsils in children is very rare and may sometimes be confused with recurrent tonsillitis. The patient had a unilateral tonsillar mass with enlarged neck lymph nodes, which necessitated radiographic evaluation and biopsy. This led to an early surgical intervention and chemotherapy.

## 1 Introduction

Histiocytic sarcoma is a rare hematological neoplasm defined by malignant cell growth that resembles mature histiocytes in appearance and immunohistochemical properties. It is loosely classified as a tumor of macrophage–dendrocyte cell lineage [1]. HS is an extremely rare tumor, accounting for less than 1% of hemato‐lymphoid cancers. The age distribution of HS is scattered, occurring predominantly in males [2]. Its occurrence in children, particularly with tonsillar involvement, is highly unusual.

Histiocytic sarcoma is diagnosed at a very advanced stage with abysmal prognoses, limited response to chemotherapy, and a high rate of mortality [3]. The overall survival (OS) rate for HS is close to 2 years [4].

Pediatric cases of HS have been reported with variable presentations and prognoses [3, 5]. Although tonsillar involvement has been reported in rare instances, these cases have predominantly been described in adults [6, 7]. Pediatric involvement of the palatine tonsil remains exceedingly uncommon, with very limited data available in the literature.

A comprehensive literature search was conducted in PubMed, Scopus, and Web of Science from database inception through January 2026 using the search terms “histiocytic sarcoma,” “pediatric,” “child,” “tonsil,” and “palatine tonsil.” Only English‐language articles were reviewed. Although isolated cases of tonsillar histiocytic sarcoma have been reported in adults, no previously published cases describing primary palatine tonsillar histiocytic sarcoma in a pediatric patient were identified. To the best of our knowledge, this represents the first reported case of pediatric primary tonsillar histiocytic sarcoma.

This case highlights the diagnostic challenges and multidisciplinary approach required in the management of rare malignancies, such as histiocytic sarcoma, especially in pediatric patients. Further follow‐up is ongoing to monitor the patient's response to chemotherapy and long‐term outcomes.

## 2 Case History / Examination

**FIGURE 1:**
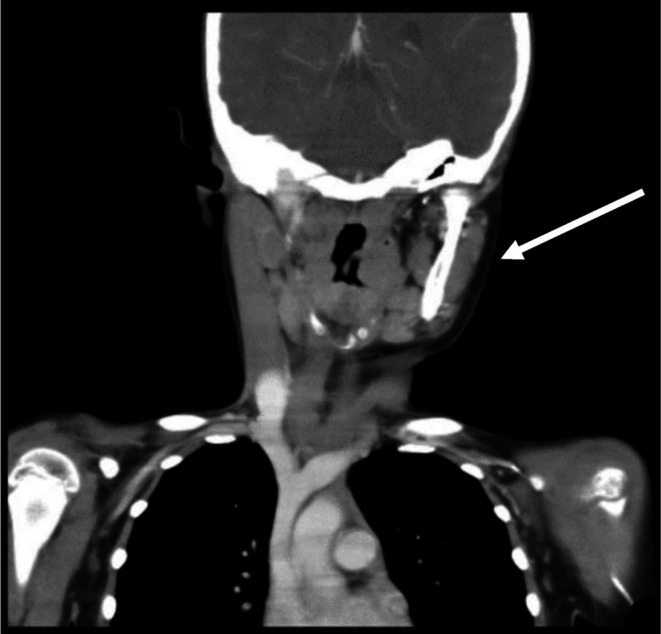
Coronal CT view demonstrating left tonsillar mass with associated ipsilateral cervical lymphadenopathy.

**FIGURE 2:**
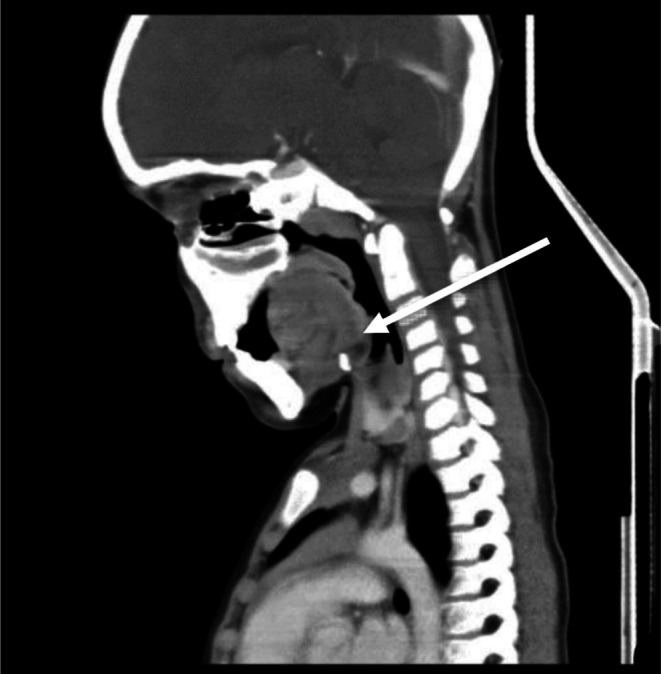
Axial CT image highlighting enlarged left cervical lymph node.

We present a 7‐year‐old boy with a history of recurrent tonsillitis, neck swelling, and foreign body sensation in the throat. On examination, an ulcerative lesion was observed on the left tonsil, and left cervical lymphadenopathy. A contrast‐enhanced CT scan showed diffusely enlarged edematous heterogeneously enhancing left palatine tonsil resulting in asymmetrical luminal narrowing with ipsilateral cervical lymphadenopathy, as shown in Figures 1 and 2.

The patient had experienced recurrent tonsillitis for approximately 6 months prior to presentation. Worsening throat pain and progressive neck swelling were noted over a 3‐week period, prompting medical evaluation. Imaging and biopsy were performed within 2 weeks of presentation, and surgical intervention followed shortly thereafter. Chemotherapy was initiated within 2 weeks after surgery.

## 3 Investigations and Treatment

**FIGURE 3:**
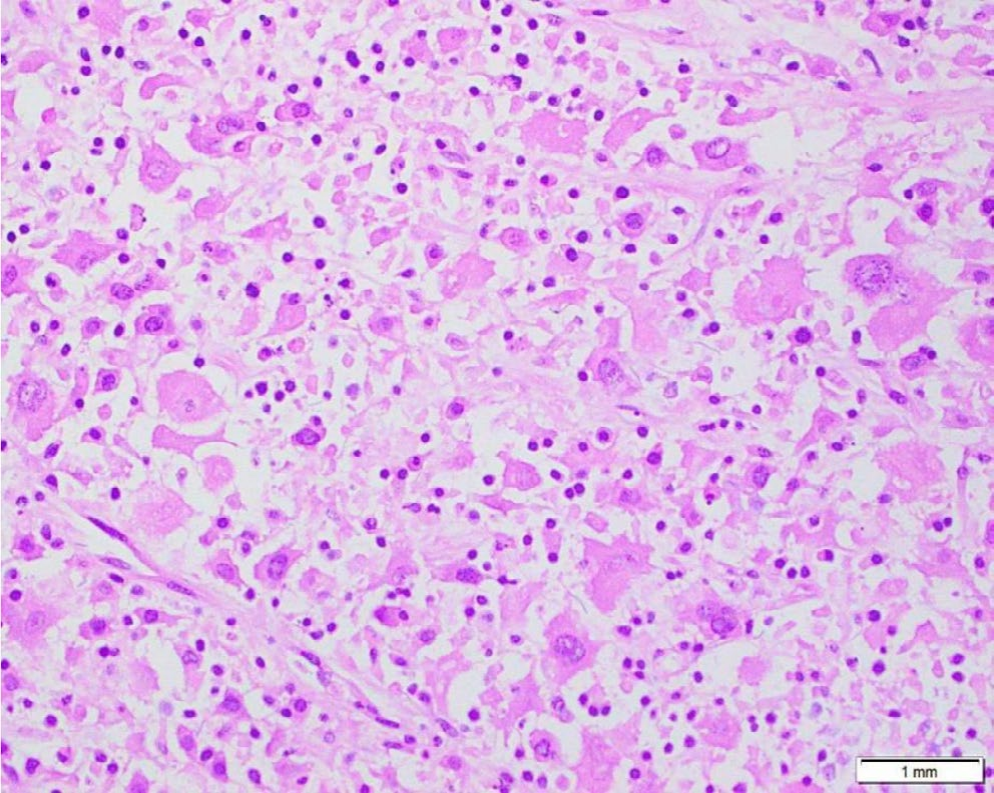
Histopathology of tonsillar mass. H&E staining (40×) showing diffuse sheets of large pleomorphic cells with abundant eosinophilic cytoplasm.

**FIGURE 4:**
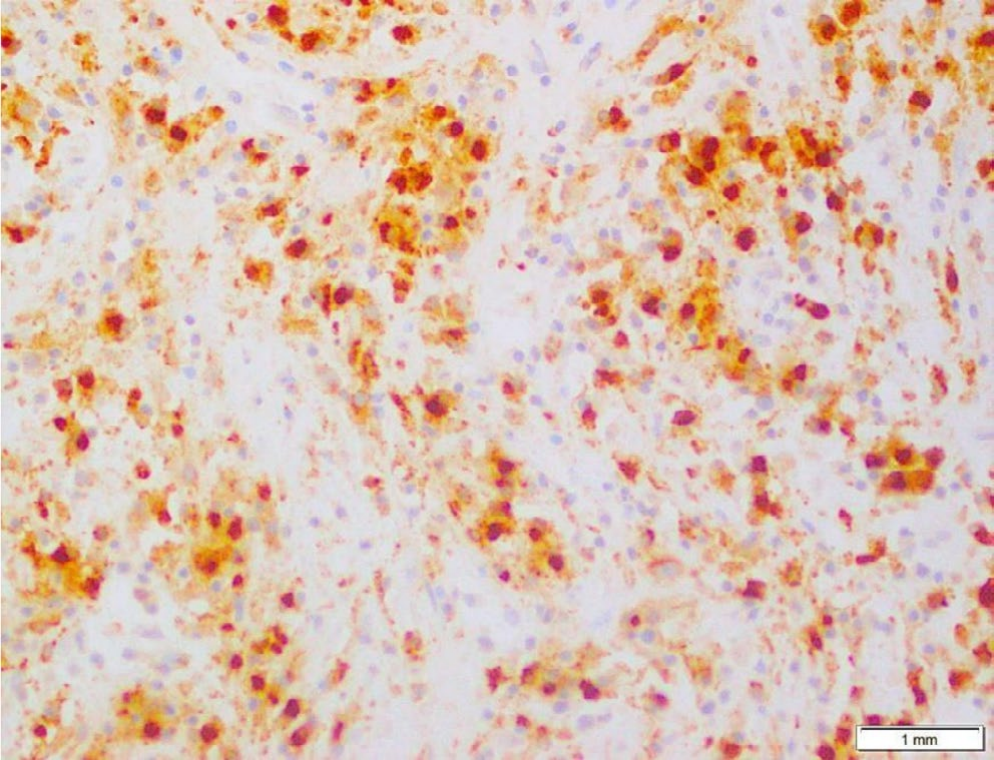
Immunohistochemistry demonstrating strong cytoplasmic positivity for CD68 and CD163 (100×). Ki‐67 immunostaining reveals a high proliferative index of approximately 80%.

A biopsy specimen was sent for immunohistopathology which showed squamous cell mucosa with underlying sheets of large round to oval cells with abundant eosinophilic cytoplasm. The nuclei were oval‐intended, grooved, and irregularly folded with vesicular chromatin and distinct nucleoli. The immunohistochemistry showed that the cells were S100 positive, raising suspicion for histiocytic sarcoma. Immunohistochemical analysis confirmed the diagnosis of HS, with the tumor cells expressing cathepsin, LCA, CD68, CD163, and CD4. The Ki‐67 proliferation index was notably high at 80%, thus confirming the diagnosis of histiocytic sarcoma, as shown in Figures 3 and 4. Tumor cells were negative for CD3, CD20, CD30, and ALK, helping exclude lymphoid and anaplastic large cell lymphoma.

The patient was referred to the otorhinolaryngology (ENT) department for further management. Surgical intervention included excision of the enlarged, ulcerated left tonsil along with the affected cervical lymph nodes. Postoperatively, the patient was started on a chemotherapy regimen consisting of cyclophosphamide (750 mg), doxorubicin (50 mg on Day 1), vincristine (1.4 mg on Day 1), and prednisolone (1 mg on Days 1–5). Aprepitant (125 mg) was also prescribed as an antiemetic. The chemotherapy cycle was planned to be repeated every 21 days.

The treatment plan was decided after multidisciplinary discussion between ENT, oncology, and pathology teams. As imaging showed localized tonsillar disease with regional lymph node involvement and no evidence of distant spread on CT, surgical excision was performed for both diagnosis and local control. A CHOP‐based chemotherapy regimen was started due to its reported use in aggressive hematologic malignancies and its tolerability in pediatric patients. Other regimens were considered; however, in the absence of established pediatric guidelines, a lymphoma‐type approach was preferred. PET‐CT and molecular testing were not performed due to resource limitations. Radiotherapy was discussed but deferred because of the patient's young age and the plan for systemic chemotherapy.

## 4 Conclusion and Results

The patient completed three cycles of chemotherapy and has been followed for 7 months to date. At the most recent follow‐up, he remains clinically stable with no evidence of recurrence. No treatment‐related complications were observed. He continues to undergo routine clinical and radiologic surveillance as per standard follow‐up protocols.

## 5 Discussion

Histiocytic sarcoma (HS) is an exceptionally rare malignancy derived from mature histiocytes, accounting for less than 1% of hemato‐lymphoid neoplasms [2]. According to the revised classification of histiocytic disorders, it is categorized as a tumor of macrophage–dendritic cell lineage [1]. Although HS is more commonly reported in adults, typically in the sixth decade of life [2, 4], pediatric cases are distinctly uncommon. Primary involvement of the palatine tonsil is particularly rare, with only a few cases documented in the literature, most of which involve adults [6, 7]. The occurrence of this entity in a 7‐year‐old child therefore represents an unusual and diagnostically challenging presentation.

Tonsillar disease in children is overwhelmingly benign. Unilateral enlargement is frequently attributed to recurrent tonsillitis, reactive hyperplasia, or peritonsillar infection. As highlighted in reports of extranodal HS, clinical manifestations are often nonspecific and may closely resemble inflammatory conditions [8, 9]. In our patient, a prior history of recurrent tonsillitis initially contributed to a benign working impression. However, the persistence of unilateral enlargement, ulceration, and associated cervical lymphadenopathy prompted further evaluation. This case underscores an important pediatric diagnostic pitfall: when common symptoms persist or evolve atypically, reconsideration of the diagnosis becomes essential.

Radiologic imaging demonstrated asymmetric enlargement of the left palatine tonsil with ipsilateral cervical lymphadenopathy. Although imaging was valuable in delineating the extent of disease, it could not reliably distinguish between chronic inflammation and malignancy, as previously noted in extranodal HS presentations [9]. Ultimately, definitive diagnosis depended on early tissue sampling. Prompt biopsy allowed histopathologic and immunohistochemical confirmation without significant delay.

Microscopically, the tumor demonstrated sheets of large pleomorphic cells with abundant eosinophilic cytoplasm and irregular nuclei, consistent with the described morphologic spectrum of HS [8, 10]. Immunohistochemical analysis showed strong expression of histiocytic markers, including CD68 and CD163, along with LCA and S100 positivity, findings that are considered characteristic for histiocytic differentiation [11, 12]. The Ki‐67 proliferation index was markedly elevated at approximately 80%, reflecting the aggressive biological potential of the tumor.

Compared with previously reported tonsillar HS cases, which often present at advanced or disseminated stages [6, 7], our patient was diagnosed at a localized stage. Early biopsy played a central role in avoiding prolonged diagnostic delay and enabled timely multidisciplinary management involving otorhinolaryngology, pathology, and pediatric oncology teams. The patient underwent surgical excision followed by systemic chemotherapy. At 7 months of follow‐up, after completing three cycles of chemotherapy, the disease remains stable without evidence of progression. Although longer surveillance is required, this early stability suggests that prompt recognition and coordinated treatment may help achieve disease control in localized pediatric cases.

HS is generally considered an aggressive malignancy with variable outcomes depending on disease extent and patient‐related factors. In a large Japanese cohort of histiocytic and dendritic cell neoplasms, Shimono et al. reported a median overall survival of 23.5 months and a 2‐year overall survival rate of 49.2%. Importantly, elevated lactate dehydrogenase (LDH), poor performance status (ECOG 2–4), and advanced Ann Arbor stage (III–IV) were identified as independent adverse prognostic factors [4]. These findings suggest that prognosis is strongly influenced by disease burden and functional status at presentation. In contrast, disseminated or high‐risk disease has been associated with considerably shorter survival in other reports [13]. Overall, outcomes appear to be heterogeneous, with localized disease potentially achieving more favorable short‐term control when managed with a multimodal approach [10, 14].

Given its rarity and its ability to closely mimic benign tonsillar pathology, histiocytic sarcoma should be considered in the differential diagnosis of persistent, asymmetric, ulcerated, or non‐resolving tonsillar lesions in pediatric patients. Our case reinforces a practical clinical message: when tonsillar findings deviate from the expected course of common inflammatory disease, early biopsy is not only justified but essential. Timely histologic confirmation enables appropriate multidisciplinary intervention and may improve disease control in this rare but aggressive malignancy.

## 6 Conclusion

The rarity of pediatric tonsillar HS and the importance of early detection via histology and immunohistochemistry are highlighted in this case. A multimodal therapy strategy is crucial because of its aggressive nature and dismal prognosis. To enhance survival results for pediatric HS patients and optimize treatment methods, more research is required.

## Author Contributions

**Ihsan ullah:** conceptualization, investigation, supervision, writing – review and editing. **Muhammad Sanaan Noor:** conceptualization, investigation, writing – review and editing. **Hammad Saif:** conceptualization, investigation, visualization. **Muhammad Maaz Bin Zahid:** conceptualization, investigation, writing – original draft. **Muhammad Hassan Noor:** methodology, validation. **Muhammad Asim Shah:** methodology, validation, writing – original draft. **Aizaz Anwar Khalid:** data curation, validation. **Somaiya Ahmed:** supervision, visualization.

## Funding

The authors have nothing to report.

## Consent

Informed written consent from the parents of the patient was obtained for the conduct of the study and the publication.

## Conflicts of Interest

The authors declare no conflicts of interest.

## Data Availability

All relevant data supporting the findings of this case report are included within the manuscript and accompanying figures. No additional datasets were generated or analyzed during the current study.
